# Percutaneous endoscopic lumbar discectomy for L5-S1 disc herniation based on image analysis and clinical findings: A retrospective review of 345 cases

**DOI:** 10.1097/MD.0000000000032832

**Published:** 2023-02-03

**Authors:** Shih-Chieh Shen, Hung-Chieh Chen, Hsi-Kai Tsou, Ruei-Hong Lin, Yu-Tung Shih, Chih-Wei Huang, Chien-Lun Tang, Hsien-Te Chen, Chien-Chun Chang, Chung-Yuh Tzeng

**Affiliations:** a Department of Surgery, Tri-Service General Hospital Songshan Branch, National, Defense Medical Center, Taiwan, R.O.C; b Department of Biomedical Engineering, National Yang Ming Chiao Tung University, Taiwan, R.O.C; c Department of Radiology, Taichung Veterans General Hospital, Taichung, Taiwan, R.O.C; d School of Medicine, National Yang Ming Chiao Tung University, Taipei, Taiwan, R.O.C; e Functional Neurosurgery Division, Neurological Institute, Taichung Veterans General Hospital, Taichung, Taiwan, R.O.C; f Department of Rehabilitation, Jen-Teh Junior College of Medicine, Nursing and Management, Miaoli County, Taiwan, R.O.C; g Department of Post-Baccalaureate Medicine, College of Medicine, National Chung Hsing University, Taichung, Taiwan, R.O.C; h College of Health, National Taichung University of Science and Technology, Taichung, Taiwan, R.O.C; i Department of Neurosurgery, Jen-Ai Hospital, Taichung, Taiwan, R.O.C; j Institute of Biomedical Sciences, National Chung Hsing University, Taichung, Taiwan, R.O.C; k Department of Neurosurgery, Neurological Institute, Taichung Veterans General Hospital, Taichung, Taiwan, R.O.C; l Department of Sports Medicine, College of Health Care, China Medical University, Taichung, Taiwan, R.O.C; m Department of Orthopedic Surgery, China Medical University Hospital, China Medical University, Taichung, Taiwan, R.O.C; n Spine Center, China Medical University Hospital, China Medical University, Taichung, Taiwan, R.O.C; o Department of Orthopedics, Taichung Veterans General Hospital, Taichung, Taiwan, R.O.C; p Department of Medicinal Botanicals and Health Applications, Da-Yeh University, Changhua County, Taiwan, R.O.C.

**Keywords:** calcified disc, lumbosacral transitional vertebrae, pediatric lumbar disc herniation, percutaneous endoscopic lumbar discectomy, ruptured disc

## Abstract

The effect of spinal anatomical anomalies on the efficacy of percutaneous endoscopic lumbar discectomy (PELD) for disc herniation repair is unclear. This retrospective review aims to assess the safety and effectiveness of PELD for treating L5-S1 disc herniation with a range of characteristics and to determine the prevalence of lumbosacral transitional vertebrae (LSTV) anatomical anomalies to facilitate pre-surgical planning. From July 2005 to June 2019, 345 patients with L5-S1 disc herniations were treated with PELD. The primary outcome was 1-year postoperative visual analogue scale scores for low back and lower limb pain. The secondary outcomes included the surgical approach used, lumbosacral bony anomalies, presence of a ruptured disc or severely calcified disc, pediatric lumbar disc herniation, recurrent disc herniation management, and the long-term outcome. visual analogue scale scores for most patients were significantly improved after surgery. The prevalence of LSTVs was 4.05% (14/345 patients) in lumbar sacralization and 7.53% (26/345 patients) in sacral lumbarization. The prevalence of ruptured and severely calcified discs was 18.55% (64/345) and 5.79% (20/345), respectively. The prevalence of pediatric lumbar disc herniation was 2.02% (7/345). The recurrence rate was 4.34% (15/345). Two durotomy cases without sequelae and 8 cases of lower limb dysesthesia lasting longer than 3 months postoperatively were reported. PELD is safe and effective for treating L5-S1 disc herniation, including cases complicated by calcified lumbar disc herniation, disc rupture with migration, and the presence of LSTV. Appropriate imaging is essential to identify case-specific factors, including the prevalent LSTV anatomical anomalies, before surgery.

## 1. Introduction

Percutaneous endoscopic lumbar discectomy (PELD) is a minimally invasive treatment for lumbar disc herniation (LDH). The benefits of PELD over open lumbar discectomy include less intraoperative blood loss, better paravertebral muscle preservation, and shorter hospital stays. Because PELD results in less epidural scarring by preserving the ligamentum flavum,^[1,2]^ disc re-treatment is safer and more effective. PELD is also proven safe and effective for the treatment of pediatric LDH.^[3,4]^ Thus, PELD has become an alternative to conventional open discectomy for LDH management. For the treatment of LDH, PELD can be performed using a transforaminal (TF) or interlaminar (IL) approach. Appropriate surgical planning, including the choice of approach, is informed by case-specific characteristics of the lesion and contributes to successful PELD outcomes for LDH. Such characteristics include the presence of lumbosacral transitional vertebrae (LSTV), the direction of disc material migration in the herniated disc, and the presence of calcified LDH.

Present in 4% to 30% of the general population,^[5,6]^ LSTV are common congenital vertebral anomalies that range in morphology from broadened transverse processes to complete fusion.^[6]^ Preoperative detection of an LSTV is essential to determining the correct spinal level for surgery and to avoiding procedural and surgical errors. Anomalous articulation or fusion between LSTV and the sacrum makes the TF approach difficult; thus, the spinal level should be identified preoperatively. In addition, disc rupture involves extrusion of the disc nucleus out of the annulus, into the spinal canal, commonly causing leg pain. Ruptured disc migration refers to disc material displacement away from the open annulus where the disc has extruded.^[7]^ Upward or downward migration should be identified during preoperative planning so that surgical management can be adjusted accordingly. Finally, calcified LDH is rare and is most prevalent in China.^[8]^ The hard texture of a calcified disc makes the TF approach difficult, requiring the IL approach in such cases. Postoperative lower limb dysesthesia seems to be more common in cases involving a calcified disc. Thus, calcified LDH identified preoperatively requires careful consideration in the surgical plan.

The purpose of this retrospective review is to assess the safety and effectiveness of PELD for treating L5-S1 disc herniation with a range of characteristics and to determine the prevalence of LSTV anatomic anomalies to assist medical professionals with pre-surgical planning. The use of different surgical techniques to accommodate case-specific disc herniation characteristics is also discussed.

## 2. Materials and methods

### 2.1. Patient demographics

We retrospectively reviewed the data of 345 consecutive patients treated with PELD for L5-S1 disc herniation at Taichung Veterans General Hospital between July 2005 and June 2019. The patient age, sex, and diagnosis were extracted from the medical records. Preoperative radiographs (anteroposterior and lateral) and magnetic resonance image (MRI) of the lumbar spine were also evaluated. Computed tomography (CT) of the lumbar spine was carried out if a calcified disc was suspected. Thoracic spine radiography (anteroposterior) was used to detect the presence and lumbosacral level of LSTV. To evaluate the effectiveness of PELD, preoperative and postoperative visual analogue scale (VAS) pain scores were recorded for each patient with low back and lower limb pain. Complications arising from PELD included cerebrospinal fluid leakage, wound infection, postoperative sequelae, and recurrent disc herniation.

This study was approved by the institutional review board of Taichung Veterans General Hospital (#CE19352B) and performed in accordance with the Declaration of Helsinki. Due to the retrospective nature of this study, informed consent (written/verbal) of the patients was waived.

### 2.2. Surgical technique

All PELD procedures were performed by the same neurosurgeon. All patients were given prophylactic antibiotics (1000 mg cefazolin sodium or 2000 mg cefazolin sodium if body weight > 80 kg) within 30 minutes of surgery. In those patients treated with the IL approach, surgery was performed with the patient under intravenous (IV) anesthesia but clearly conscious and lying prone on a radiolucent table.^[9]^ The skin incision was made as close as possible to the medial area of the craniocaudal center of the IL window, facilitated by insertion of a 2-channel dilator into the lateral edge of the IL window. A working sleeve with an 8.0 mm outer diameter and beveled opening was then directed toward the ligamentum flavum. The remainder of the procedure was performed under direct visual control and with constant irrigation. A lateral incision window (6–8 mm) was made in the ligamentum flavum to expose the neural structures and epidural fat tissue. The working sleeve with beveled opening could be turned and used as a nerve hook. Using the joystick principle, medial and lateral as well as cranial and caudal mobility within the spinal canal could be manipulated to search for and remove the protruding disc with the aid of control optics and a bipolar probe. All surgical instruments and the endoscopic system were supplied by Richard Wolf GmbH (Knittlingen, Germany). The high-resolution endoscope had a 4.1 mm intra-endoscopic working channel, and the direction of view was 25°. The combination of the 8.0-mm outer diameter and beveled opening of the working sleeve enabled us to create visual and working fields in an area without a clear, anatomically pre-formed cavity. A high radiofrequency low temperature bipolar probe (Elliquence, LLC, Baldwin, NY) was used.

Based on preoperative MRI findings, the IL axillary or shoulder approach was chosen depending on the location of the disc herniation and the presence or absence of migration. For patients treated with the TF approach, surgery was performed under IV anesthesia, with the patient clearly conscious and lying prone on a radiolucent table. The insertion site was localized at the highest laminal level of the symptomatic side using the fluoroscopic lateral view. A spinal cannula was gently inserted into the selected disc at the dorsal aspect. A guide wire was then placed along the spinal cannula, and the spinal cannula then was removed. A skin incision about 8 mm in length was made and a 2-channel dilator inserted into the disc space along the guide wire. The working sleeve and endoscope were then inserted as previously described. Under direct visual control, the bulging disc was removed and the posterior annulus fibrosus adequately decompressed.

**Figure 1.**
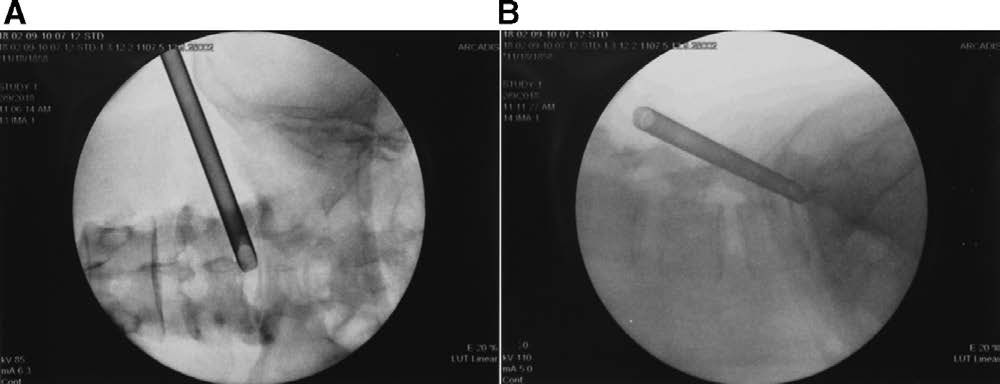
The transforaminal approach on the L5-S1 level was used to indicate the lower iliac crest condition. (a, b) A 41-years-old female presented with low back pain and painful numbness radiating to the left buttock and a history of more than 1 year of left lower limb pain.

All patients treated with the TF approach had a low iliac crest or LSTV, eliminating the need for foraminoplasty (Fig. 1). Among our patients, the TF approach was used in 9.27% (32/345), the IL axillary approach in 72.75% (251/345), and the shoulder approach in 18.26% (63/345). One patient with a huge bulging disc underwent both the axillary and shoulder approaches.

### 2.3. Outcome assessment

The primary outcome was the mean 1-year post-PELD VAS score for low back and low limb pain. The secondary outcomes included patient satisfaction, the site of disc herniation, LSTV, migration of ruptured disc herniation, severe calcified disc herniation, surgical approach, and pediatric disc herniation. Adverse events including complications from PELD were recorded. The MacNab scale score was used as the indicator of clinical outcomes and pain relief.^[10]^ The MacNab approach classifies clinical symptoms into 4 groups (excellent, good, fair, and poor), but this score was not accurate enough to classify pain. Thus, we modified the MacNab score to include the VAS score (post-PELD 1-year VAS compared with pre-PELD VAS) in the clinical outcomes for the MacNab score. The modified MacNab score classified pain as follows: excellent (no pain; VAS = 0 and 100% pain relief); good (50–99% pain relief), fair (1–49% pain relief), and poor (0% pain relief and no overall improvement).

### 2.4. Statistical analysis

All data are presented as the mean and standard deviation (SD). All analyses were conducted using Microsoft Excel 2016 software (Microsoft, Inc, Redmond, WA).

## 3. Results

**Figure 2.**
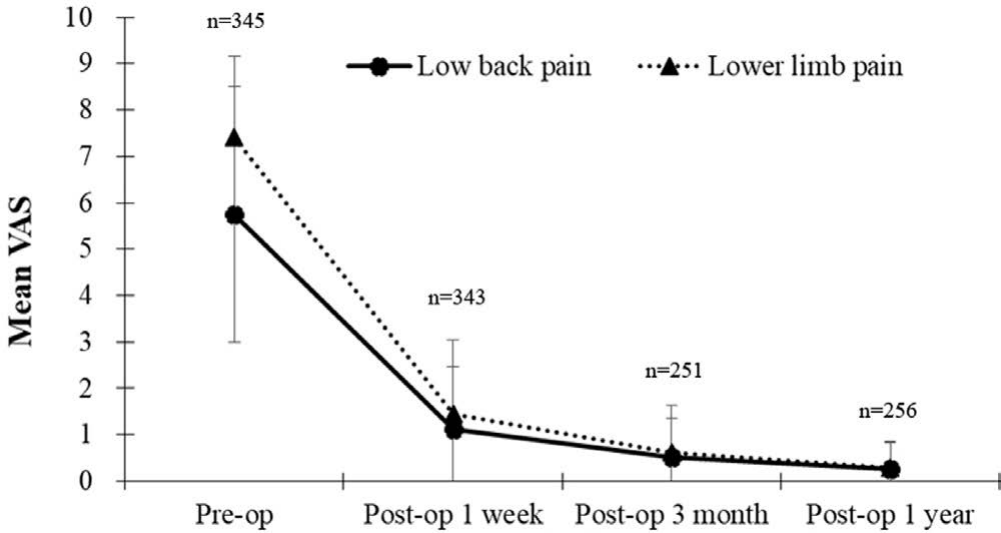
The number of patients followed and the mean VAS score according to the medical record before surgery, then at 1 week, 3 months, and 1-year post-surgery. The line graph showed VAS scores for low back pain and lower limb pain, respectively, in the follow-up period. VAS = visual analogue scale.

The patient cohort included a total of 345 patients (219 males; 126 females) with a mean age of 39.40 years (range, 13–84 years) (Table 1). The mean VAS scores for low back pain and lower limb pain decreased dramatically from baseline within 1 week after surgery and continued to decrease through the following year of follow up (Table 2; Fig. 2). Of the 256 patients followed for a year, the mean low back pain VAS score decreased from 5.75 (± 2.75) preoperatively to 0.26 (± 0.57) 1-year post-PELD. The mean lower limb pain VAS score decreased from 7.41 (± 1.74) preoperatively to 0.29 (± 0.58) 1-year post-PELD (Table 2; Fig. 2). Excellent outcomes on the modified MacNab scores^[10]^ were reported by 60.14% of the cohort for low back pain and 72.69% for lower limb pain. Good outcomes were reported by 33.94% for low back pain and 21.40% for lower limb pain. Nearly 4% had a fair outcome for relief of low back and lower limb pain. A poor outcome was reported by 2.21% for low back and lower limb pain. Fifteen patients required repeated surgery.

**Table 1:** Baseline characteristics of the study cohort of 345 patients.

	Number of patients
Male/Female	219/126
Age (yr)	39.40 (range, 13–84)
Mean preoperative VAS score (low back pain/lower limb pain)	5.75/7.41
Surgical approach*	
Interlaminar axillary	251 (72.75%)
Interlaminar shoulder	63 (18.26%)
Transforaminal	32 (9.27%)
Site of disc herniation	
Left	212 (61.44%)
Right	133 (38.55%)
LSTVs	
Lumbar sacralization	14 (4.05%)
Sacral lumbarization	26 (7.53%)
6th lumbar vertebra	3 (0.86%)
Ruptured disc herniation**	64 (18.55%)
Upward	8 (12.5%)
Downward	27 (42.18%)
No migration	30 (46.87%)
Severe calcified disc herniation	20 (5.79%)
Pediatric disc herniation	7 (2.02%)
Recurrent disc herniation	15 (4.34%)

* One patient underwent both axillary and shoulder approaches owing to a huge bulging disc.

** One patient exhibited both large upward and large downward migrated discs.

LSTV = lumbosacral transitional vertebrae, VAS = visual analogue scale.

**Table 2:** Patient mean VAS scores before and after PELD.

	Pre-PELD	Post-PELD	Post-PELD	Post-PELD
		1 wk	3 mo	1 yr
Mean VAS score				
Low back pain	5.75	1.12	0.52*	0.26*
Lower limb pain	7.41	1.45	0.62*	0.28*
Number of patients	345	343	251*	256*

* The mean VAS at 3-month post-PELD is the mean of 251 patients and 1-year post-PELD is the mean of 256 patients; 15 patients who underwent repeated operations were excluded.

PELD = percutaneous endoscopic lumbar discectomy, VAS = visual analog scale.

**Figure 3.**
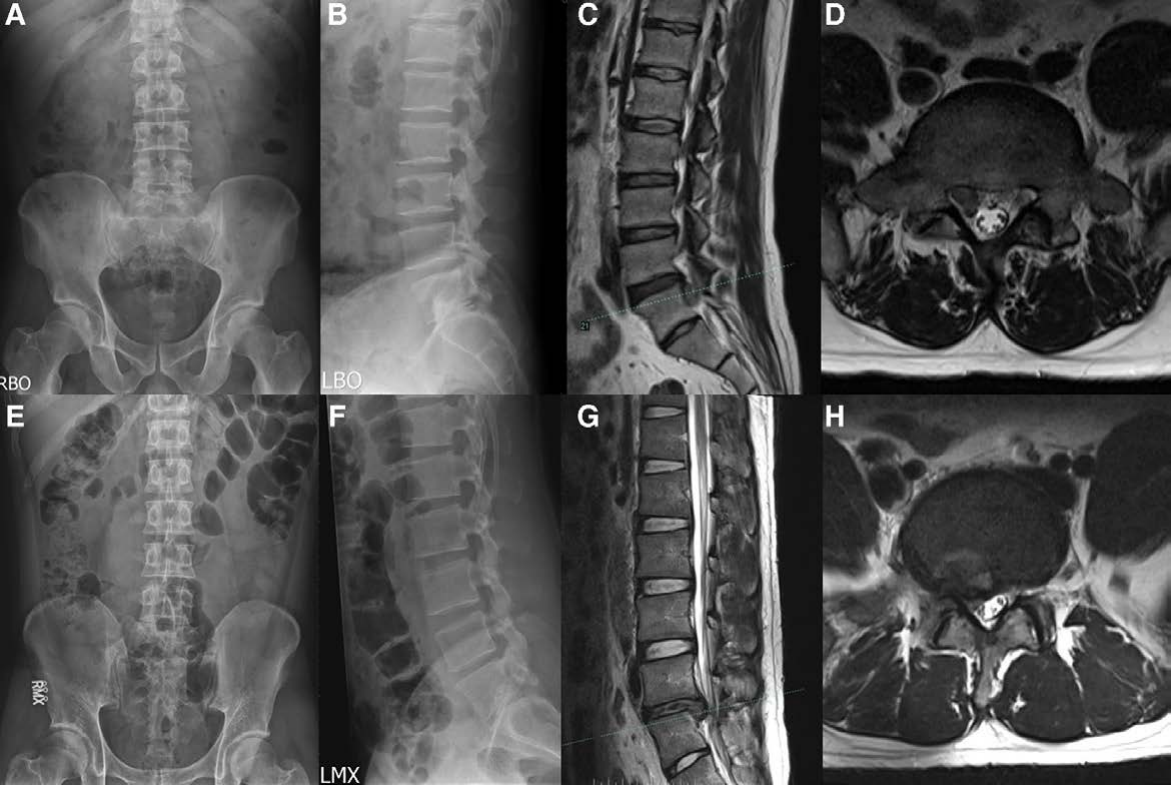
Two patients with lumbosacral transitional vertebrae. (a-d) A 36-year-old male presented with low back pain with pain radiating to the right lower limb for 3 months. (a, b) Radiographs of the lumbar spine showed L5 sacraliation. (c, d) Preoperative MRI showed L4-S1 disc herniation with downward migration of the right ruptured disc. (e-h) A 26-years-old male presented with right buttock pain radiating to the right lower limb. (e, f) Radiographs of the lumbar spine showed S1 lumbarization. (g, h) Preoperative MRI showed L5-S1 disc herniation on the right side. MRI = magnetic resonance imaging.

**Figure 4.**
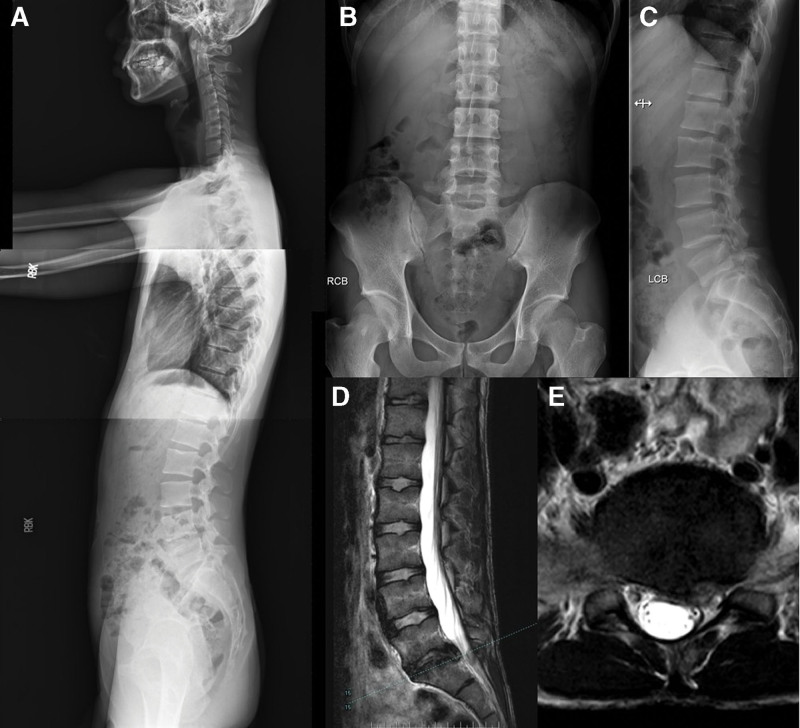
A 23-years-old male presented with low back pain and a 2-year history of painful numbness radiating to the left lower limb. (a, b, c) Radiographs of the whole spine showed the presence of the sixth lumbar vertebra. (d, e) Preoperative MRI showed L6-S1 disc herniation to the left side. MRI = magnetic resonance imaging.

**Figure 5.**
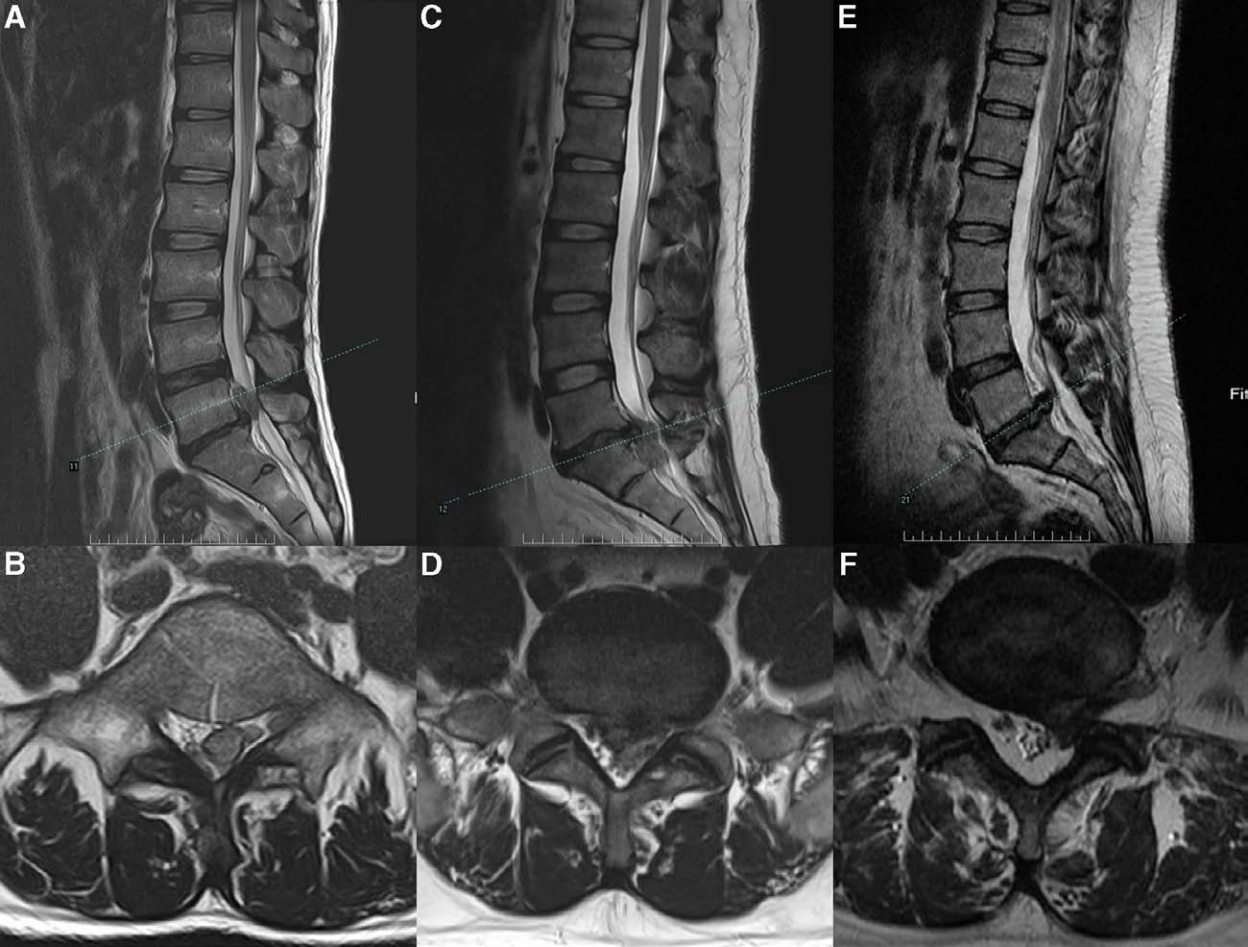
Three patients with a ruptured disc with or without migration. (a, b) A 41-year-old male with a ruptured disc and upward migration. (c, d) A 36-years-old male with a ruptured disc and downward migration. (e, f) A 37-years-old female with a ruptured disc and no migration.

**Figure 6.**
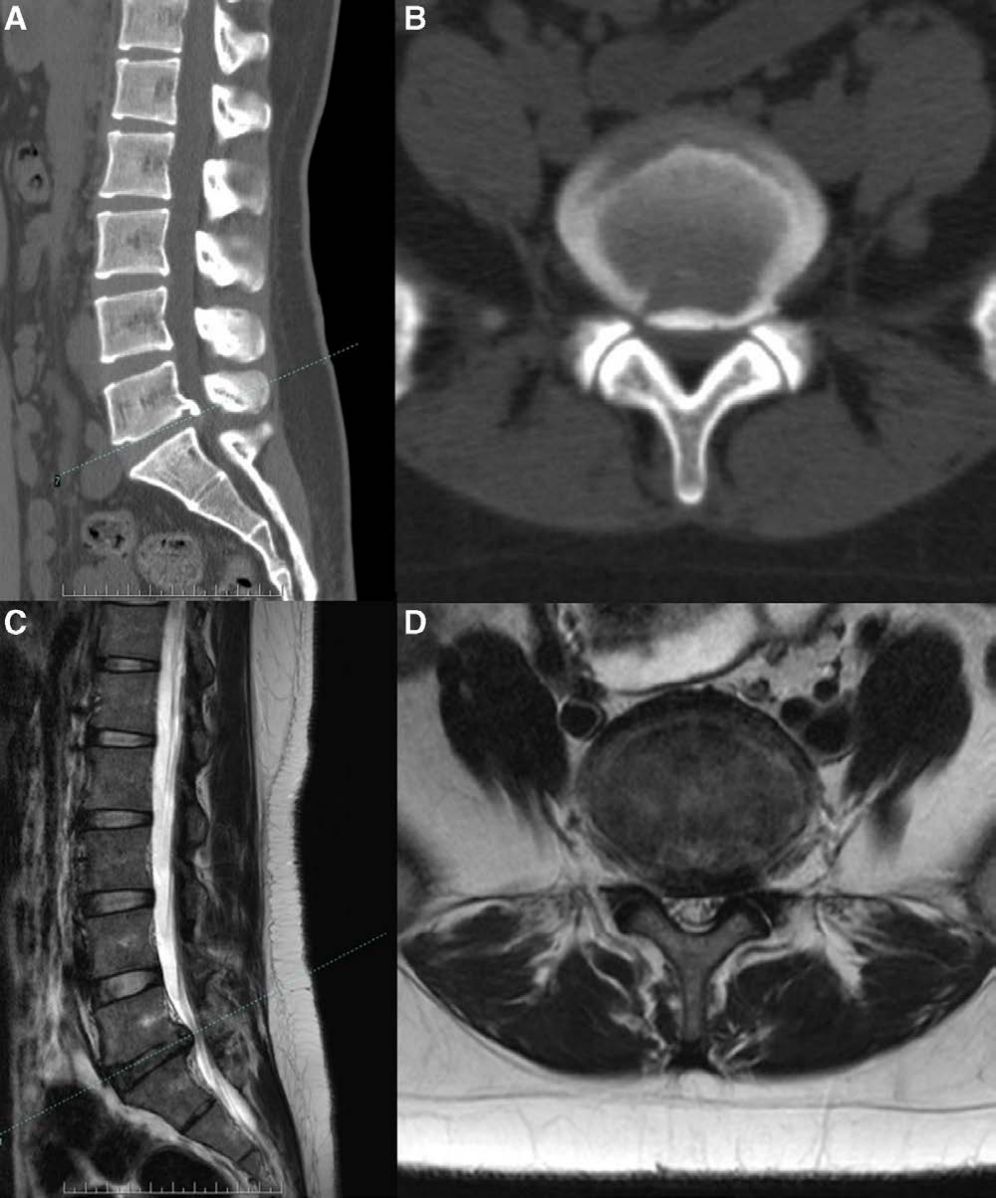
A 21-year-old female presented with low back pain and a 3-month history of painful numbness radiating to the right lower limb. (a, b) Preoperative CT showed calcified disc herniation at the L5-S1 level. (c, d) Preoperative MRI showed disc herniation dominant to the right side. CT = computed tomography, MRI = magnetic resonance imaging.

**Figure 7.**
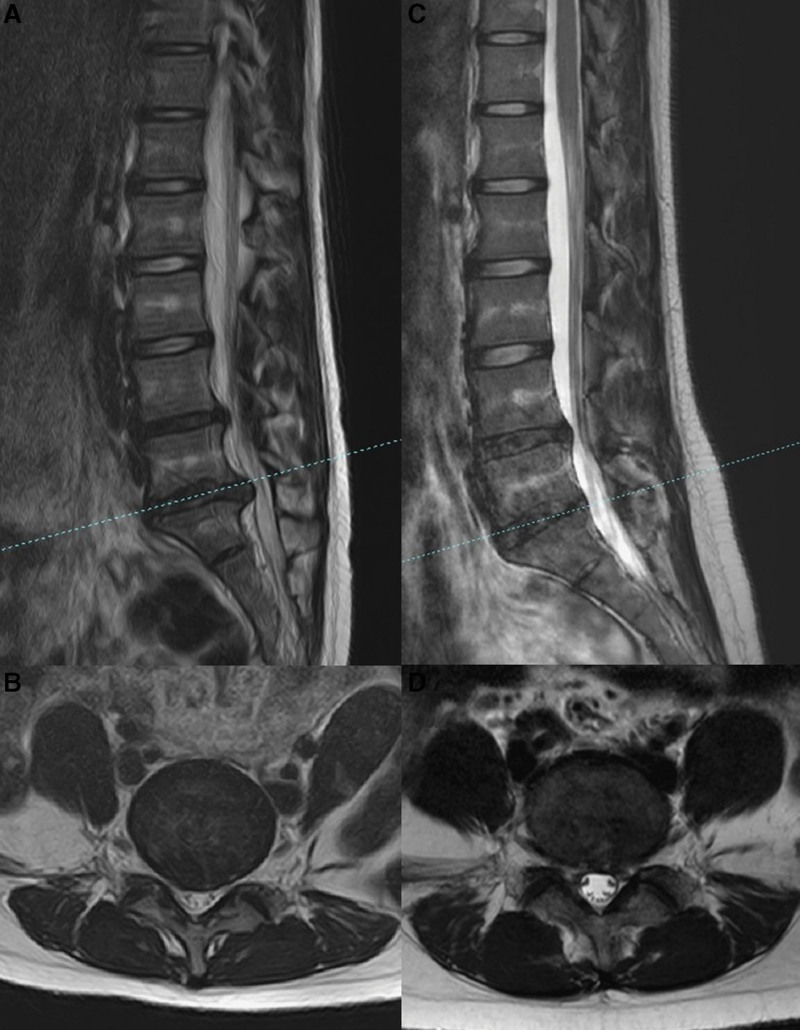
A 14-year-old girl presented with low back pain and an 8-month history of painful numbness radiating to the right lower limb. (a, b) Preoperative MRI showed disc herniation at the L4-5 and L5-S1 levels, especially at the L5-S1 level. (c, d) Postoperative MRI showed that the herniated disc had been removed, and both S1 roots were decompressed. MRI = magnetic resonance imaging.

The prevalence of specific LDH characteristics and spinal anomalies in our cohort was revealed by analysis of medical record data. Herniated discs occurred more frequently on the left side (61.44%; 212/345) than on the right (38.55%; 133/345). The prevalence of lumbar sacralization and sacral lumbarization were 4.05% (14/345) and 7.53% (26/345), respectively (Fig. 3). The prevalence of a sixth lumbar vertebra was 0.86% (3/345) (Fig. 4). The prevalence of ruptured disc was 18.55% (64/345). Migration was upward in 12.5% (8/64) of cases, downward in 42.18% (27/64), and absent in 46.87% (30/64). (Fig. 5). One patient exhibited large upward and downward disc migration. The prevalence of severe disc calcification was 5.79% (20/345) (Fig. 6). The incidence of pediatric disc herniation was 2.02% (7/345) (Fig. 7).

The rate of herniated disc recurrence was 4.34% (15/345). Ten cases of recurrent disc herniation at the same site were treated with repeated endoscopic surgery; the other 5 cases were treated with major surgery. In addition, 11 cases of recurrent herniated intervertebral disc occurring after a previous major operation were successfully treated with PELD. The characteristics of the herniated discs are detailed in Table 1.

Patient satisfaction with PELD (defined as at least 50% pain relief^[11]^) measured 1-year post-PELD was 94.09% (255/271) for low back pain and lower limb pain.

All surgeries were performed under IV sedation, and all patients were conscious during the procedures. No patients were converted to other surgical techniques. Postoperative complications included 2 durotomy cases without sequelae and no need for repairs; 8 patients had a history of lower limb dysesthesia lasting longer than 3 months. Six lower limb dysesthesia cases involved severely calcified discs, and 2 cases involved recurrent herniated discs. Thus, severely calcified discs and recurrent herniated disc surgery are risk factors for dysesthesia. No infections or surgical failures occurred.

## 4. Discussion

In this study of PELD outcomes for LDH repair in 345 patients, we observed a dramatic improvement in median postoperative VAS scores compared to preoperative scores starting 1 week after surgery with continued improvement over the next year of follow up. The LDH recurrence rate was 4.34%. Patient satisfaction was high, with a median of 94.09% at 1-year post-PELD. Our cohort included patients with LSTV anatomical anomalies (lumbar sacralization, 4.05%; sacral lumbarization, 7.53%). The prevalence of ruptured discs was 18.55% and that of severely calcified discs was 5.79%. The prevalence of pediatric lumbar disc herniation was 2.02%. These results suggest that PELD L5-S1 disc repair has excellent clinical outcomes and few surgical complications for treating LDH with a broad range of characteristics.

Previous studies have shown that PELD using either the IL or TF technique yields favorable outcomes for patients with herniated lumbar discs.^[1,9,12]^ Yeung et al reported an 89.3% satisfactory result rate among 307 patients treated by posterolateral endoscopic discectomy.^[1]^ Ruetten et al reported a 91% satisfaction rate among 331 patients treated with the endoscopic IL technique.^[12]^ Cao et al^[13]^ reported satisfaction rates of 89.8% (day surgery mode) and 91.0% (non–day-surgery mode) in their study of 402 patients. Consistent with the above reports, nearly all of our 345 patients gained immediate relief from low back pain and lower limb pain after nerve root decompression, as indicated by postoperative VAS scores. And based on the modified MacNab scores,^[10]^ around 94.09% of patients reported excellent and goods outcomes for low back and lower limb pain.

We observed that more herniated discs occurred on the left (212 patients) than on the right side (133 patients). A previous study reports that paramedian annular tears were more common on the left than on the right side.^[14]^ This difference likely reflects the fact that the right back muscles tend to be stronger because most people are right-handed. The distance from the multifidus muscle to the lamina has been shown to correlate significantly with multifidus asymmetry in patients with LDH.^[15]^ Another study, however, failed to confirm significant asymmetry in the multifidus muscle above, at, or below the disc herniation site.^[16]^ This hypothesis will require further examination.

LSTV is a congenital spinal anomaly involving sacralization of the lowest lumbar segment or lumbarization of the most superior sacral spinal segment. The reported prevalence of LSTV in symptomatic patients ranges from about 7% to 30%^[17,18]^ and was 11.59% (40/345) in our patient cohort. LSTV is of clinical significance due to the altered spinal biomechanics resulting from these anatomic anomalies. Disc degeneration and disc herniation are associated with LSTV.^[17,19]^ In younger patients, disc herniation and spondylosis are more common in those with LSTV than without.^[19,20]^ We observed no significant difference in LSTV prevalence according to age (average age of all patients, 39.40; average age of patients with LSTV, 39.32 years). Local stress caused by LSTV leads to increased disc degeneration at the transitional level and above. The observation of a smaller lordotic curve in the lumbosacral segment and a “squared” appearance of the transitional vertebrae during surgery suggests that the spinal level might have been misidentified. For accurate spinal segmentation assessment before surgery, a complete radiographic spinal survey (including T-L-S spinal x-ray films) is recommended. Anomalous articulation or bony fusion of the transverse process with the sacrum (Castellvi types II, III, and IV) can obstruct access via the TF approach; thus, such cases should be accessed using the IL approach.^[21]^

A ruptured disc displaces mainly along the horizontal and vertical planes,^[7,14]^ but the direction of disc migration observed on MRI differs between studies. In our study, downward migration of the ruptured disc at the L5-S1 level (27 cases) was more prevalent than was upward migration (8 cases). One patient exhibited equally large upward and downward migrated discs. Schellinger et al^[22]^ suggested that extruded LDH has an equal chance of displacing upward or downward because the anterior epidural space configuration is similar at both the superior and inferior vertebral bodies. Daghighi et al^[23]^ reported that caudal (72.2%) and paracentral (74.2%) migrations were the most common extruded LDH patterns noted in 1020 patients, also confirmed by Son et al^[24]^ in 164 patients with extruded LDH. Daghighi also noted that the incidence of upward migration increased with patient age and decreased in the lower lumbar spinal levels. To explain this observation, Fries et al^[25]^ hypothesized that the anterior epidural space rostral to the disc space is more available in older patients, who have less fatty tissue and whose bodies are widely patent in the higher lumbar interspace. In our study, the 7 patients with ruptured discs with upward migration only (mean age: 40.57 years) were slightly older than the patient population as a whole (mean age: 39.40 years). Surgical management and preoperative plans should be adjusted for each patient to accommodate the migration pattern of the ruptured disc.^[24]^

Calcified LDH is challenging to treat surgically, with an increased risk of incomplete decompression and nerve root injury. The reported prevalence of intervertebral disc calcification varies between studies, from rare^[26]^ to as high as 50% to 70% of patients.^[8,27]^ Because preoperative imaging studies are based on MRIs, not CTs, evaluating every patient for calcified discs can be difficult. In our study, the prevalence of severe disc calcification (defined as a disc that is primarily hard) based on preoperative CT imaging, intraoperative findings, and microscopically confirmed data, was 5.79%. Preoperative identification of disc calcification is a key step to successful treatment. The endoscopic IL approach may provide superior access to calcified herniated discs, with better visualization of the interface between the herniated disc, nerve root, and dural sac. Dural adhesion to the calcified disc is usually found during surgery. A trephine, burr, or punch used during surgery should be manipulated carefully to avoid injury to the nerve root and dural sac. Even if complete removal of the calcified disc is not possible, decompression of the affected root is essential.

Pediatric LDH, a rare condition, is treated surgically in 0.5% to 5% of patients.^[3]^ The prevalence of pediatric patients in our cohort was 2.02% (7/345). PELD is an ideal technique for pediatric LDH because of its lower level of traumatization compared to other methods. Wang et al found satisfactory outcomes with low complication rates when using percutaneous endoscopic IL discectomy in pediatric patients. Despite significant pain relief following open discectomy for pediatric LDH, long-term data suggest that 20% to 30% of pediatric patients will require additional surgery later in life.^[28]^

Recurrent disc herniation after surgical repair remains a concern. In a meta-analysis of 63 studies of PELD for LDH, the range of reported rates of recurrence was 0% to 12.5%, with an overall pooled prevalence of 3.6%.^[29]^ Similarly, 15 patients in our cohort experienced recurrent disc herniation at the same site after PELD, a rate of 4.34%. Of these patients, 10 were treated with repeated PELD. All experienced significant improvement with no major complications.

Many studies have reported a lower incidence of serious complications such as durotomy, neurological deficits, and infection with PELD than with conventional open discectomy.^[30,31]^ Injury to traversing and exiting nerve roots that results in persistent leg pain, dysesthesia, and weakness can be avoided by performing PELD under IV sedation with the patient clearly conscious. By working with patient responses, manipulation of the working sleeve can be immediately adjusted to reduce over-traction of the nerve root. Of our 345 patients, 2 had durotomy without sequelae and 8 experienced lower limb dysesthesia longer than 3 months postoperatively. No complications of cerebrospinal fluid leakage, wound infection, poor healing, or permanent neurological deficit were noted.

The primary limitation of our study is its retrospective design. We had no control group with which to perform a comparative study. We lost 74 patients (74/345; 21.44%) to follow-up 1 year post-surgery, which may have introduced statistical bias in those results. Further randomized, prospective studies are needed to verify the efficacy of PELD at the L5-S1 level, especially in pediatric patients.

## 5. Conclusions

PELD is safe and effective for treating L5-S1 disc herniation, including cases complicated by calcified LDH, disc rupture with migration, and the presence of LSTV. Appropriate imaging is essential to identify case-specific factors and anatomical anomalies before surgery.

## Acknowledgments

The authors thank Mr. Jun-Peng Chen at the Biostatistics Task Force of Taichung Veterans General Hospital for statistical analysis.

## Author contributions

**Conceptualization:** Shih-Chieh Shen, Hsi-Kai Tsou, Hsien-Te Chen, Chien-Chun Chang, Chung-Yuh Tzeng.

**Data curation:** Hung-Chieh Chen, Hsi-Kai Tsou, Yu-Tung Shih.

**Investigation:** Hung-Chieh Chen, Yu-Tung Shih.

**Methodology:** Ruei-Hong Lin.

**Project administration:** Hsi-Kai Tsou, Chih-Wei Huang, Chien-Lun Tang.

**Validation:** Ruei-Hong Lin.

**Visualization:** Hsien-Te Chen, Chien-Chun Chang.

**Writing – original draft:** Shih-Chieh Shen, Hsi-Kai Tsou.

**Writing – review & editing:** Hsi-Kai Tsou, Chung-Yuh Tzeng.
